# Hemorrhagic Cervical Synovial Cyst With Atypical Chest Pain Presentation

**DOI:** 10.7759/cureus.75178

**Published:** 2024-12-05

**Authors:** Thomas S Lee, Joseph J Lee, Allyson Resnick, Ira M Garonzik

**Affiliations:** 1 Physical Medicine and Rehabilitation, Sinai Hospital of Baltimore, Baltimore, USA; 2 Neurology and Rehabilitation Medicine, George Washington University School of Medicine and Health Sciences, Washington, D.C., USA; 3 Global Public Health and Biology, New York University College of Arts and Science, New York, USA; 4 Physical Medicine and Rehabilitation, Nova Southeastern University Dr. Kiran C. Patel College of Osteopathic Medicine, Fort Lauderdale, USA; 5 Baltimore Neurosurgery and Spine Center, Sinai Hospital of Baltimore, Baltimore, USA

**Keywords:** case report, cervical facet, cervical synovial cyst, hemorrhagic cyst, non-cardiac chest pain

## Abstract

Cervical synovial cysts are rare, especially hemorrhagic cervical synovial cysts. The patient was a 58-year-old male with a five-month history of tingling in his right shoulder region, radicular pain in his right arm, and increased pain on the right chest wall that worsened with lying supine down. The patient was diagnosed with a right-sided hemorrhagic synovial cyst at the C7-T1 level. In addition, we have a unique presentation of chest pain, when lying down from a musculoskeletal or neurological etiology instead of a cardiac etiology. The etiology of chest pain of this patient is atypical and not caused by angina. The patient underwent decompression surgery and cyst removal with total resolution of his pain and symptoms.

## Introduction

Synovial cysts in the lumbar spine are more commonly found than in the cervical spine [1,2]. Cervical synovial cysts are overall very uncommon [3-7].

Lumbar synovial cysts are more frequently reported in the literature. Cervical synovial cysts are uncommon, and hemorrhagic intraspinal synovial cysts are even rarer. To our knowledge, our case is the seventh documented case [1,3-7]. Clinical presentation would be dependent on the location, size, and adjacent structures. Acute presentation is not uncommon for documented cases. Our patient presented with symptoms over a short period without a history of trauma. Acute cases are more likely related to edema with underlying compression of neural structures, while subacute presentations may occur when there is micro-bleeding or minimal increase in the cyst volume. For hemorrhagic cysts, the typical MRI presentation is low-to-intermediate T1-weighted and low T2-weighted signal intensity [1]. However, the MRI presentation may vary based on the staging of the blood component [2].

In a previous case report and literature review by Jitpun and Narischat, the proposed pathogenesis of hemorrhagic spinal synovial cysts included degenerative zygapophysial (facet) joint changes and instability leading to a defect in the joint capsule, synovial hyperplasia secondary to inflammatory process, collagen connective tissue myxoid degeneration, and repetitive trauma [2]. Proposed risk factors for intra-cystic bleeding include trauma or microtrauma [8], anticoagulant therapy [9], and neovascularized wall of the synovial cyst [10]. A second literature review attempted something similar regarding the pathogenesis of cervical synovial cysts but not necessarily hemorrhagic cysts [11]. In a separate systematic review of lumbar intraspinal synovial cysts, they were found coincidently with spondylolisthesis, osteoarthritis, and degenerative disc degeneration in 43.4%, 40.5%, and 13.2%, respectively [12]. To date, there is no similar systematic review in the cervical spine. Xu et al. [8] performed a retrospective review of documented hemorrhagic lumbar synovial cysts in the literature and found a correlation between direct physical trauma and increased risk of hemorrhagic synovial cysts, but not all cases were associated with trauma. The reported hemorrhagic cervical synovial cyst cases do not have trauma associated with them, including our case. However, one case [3] had anticoagulant therapy but not in our presented case.

## Case presentation

History

**Figure 1:**
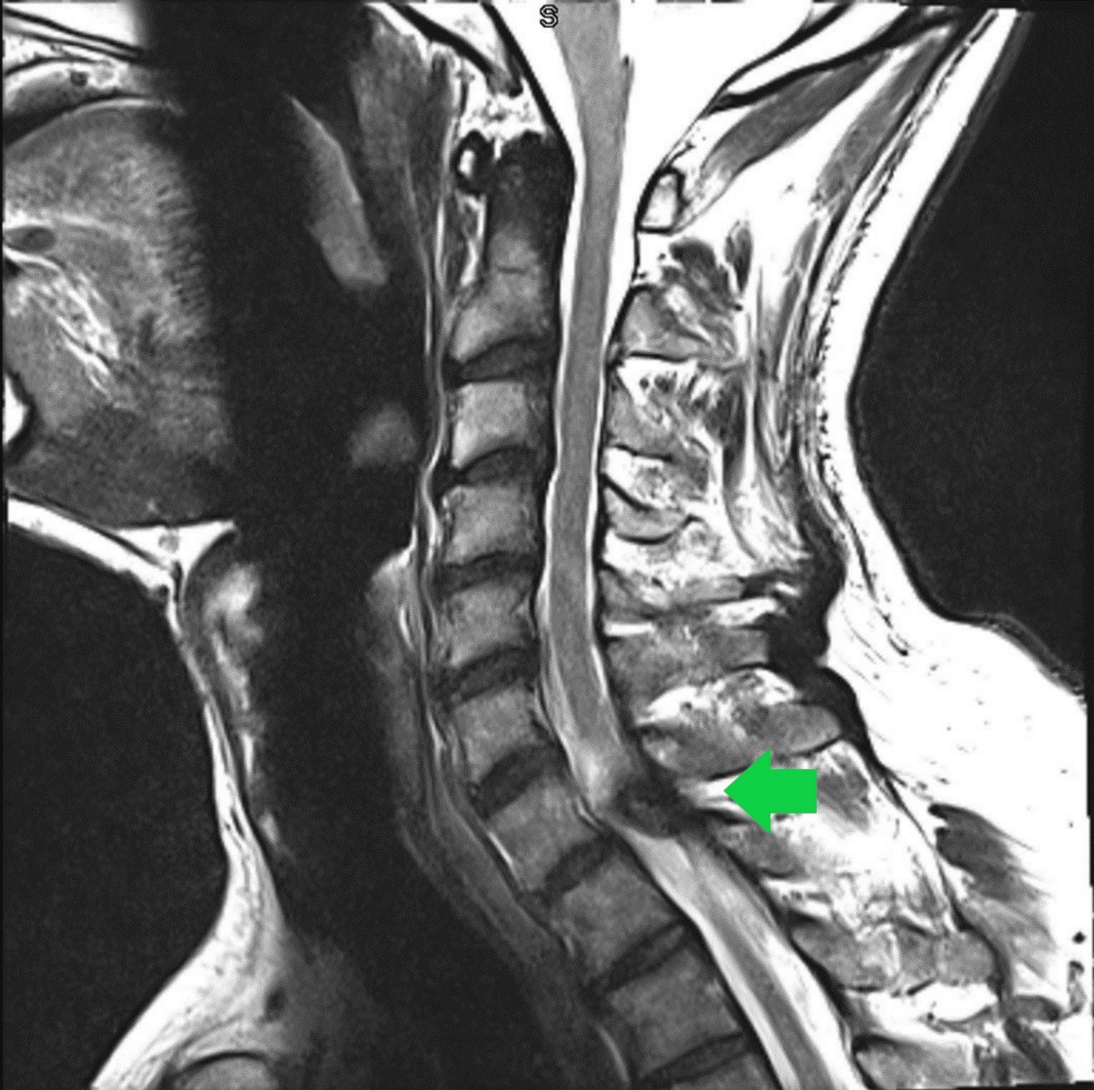
Sagittal MRI demonstrating the cyst and its relationship with the spinal cord.

**Figure 2:**
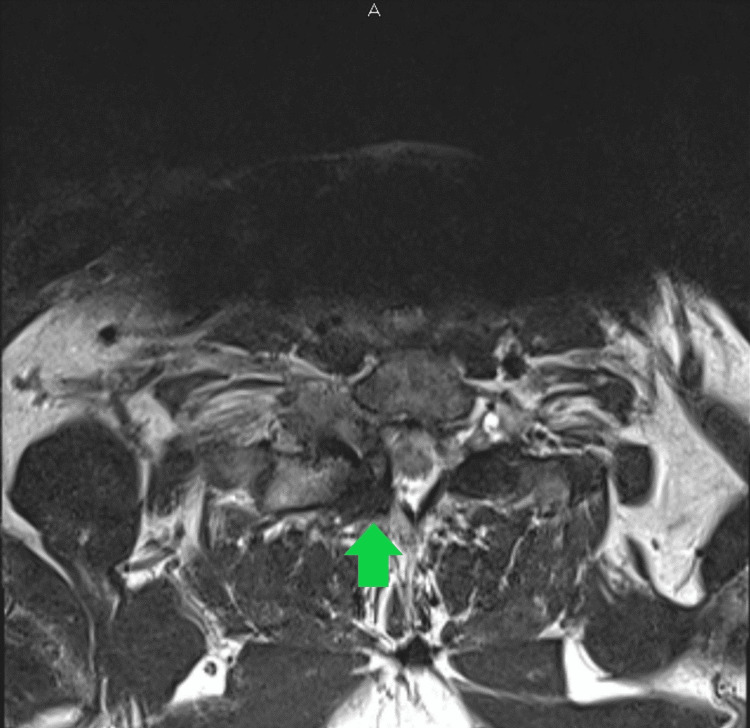
Axial MRI demonstrating the cyst and its relationship with the spinal cord.

**Figure 3:**
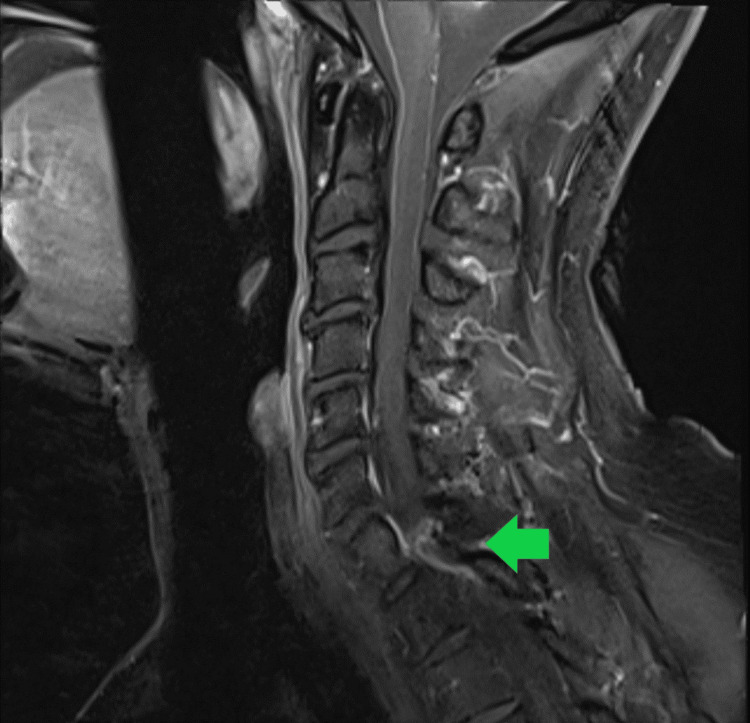
Contrast-enhanced sagittal MRI demonstrating the cyst.

**Figure 4:**
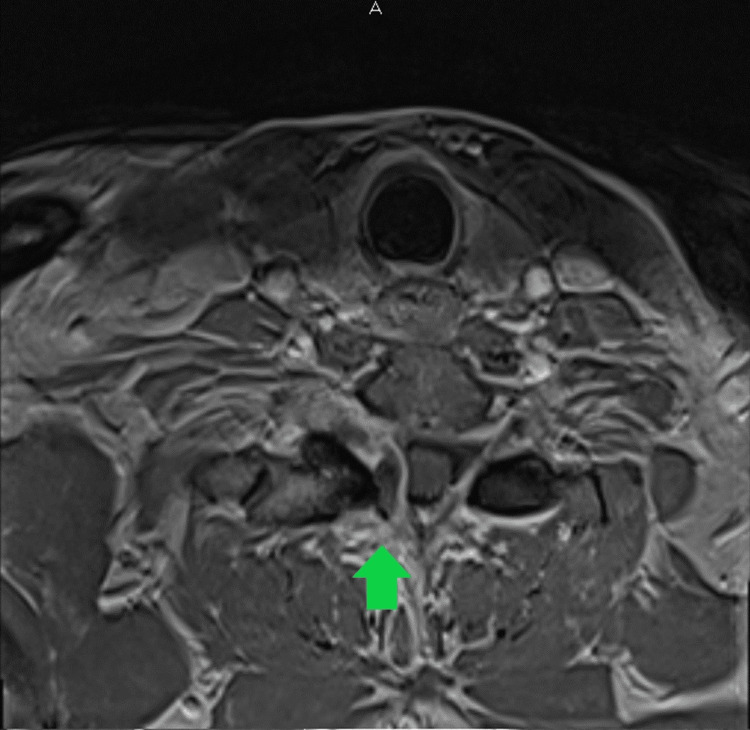
Contrast-enhanced axial MRI demonstrating the cyst.

The 58-year-old male initially experienced tingling in the right shoulder region for five months, without any precipitating event. The tingling progressed to involve the right chest wall with subsequent pain development, including the forearm and right medial hand. Oral anti-inflammatories and muscle relaxants were ineffective. A cervical spine MRI demonstrated findings consistent with a hemorrhagic synovial cyst at the right C7-T1 level, along with severe right and mild left zygapophysial degenerative changes and severe ligamentum flavum thickening, resulting in severe right foraminal stenosis. There was mild-to-moderate central canal narrowing (Figures 1-4). A cervical spine CT scan had similar findings. Cervical spine X-rays did not show any spondylolisthesis or instability. 

Six sessions of physical therapy and four sessions of chiropractic care provided no benefit. After five months of progressive symptoms, he presented for evaluation and treatment. Initially, his pain was constant and aching in the right neck, radiating into the right shoulder, anterior chest wall, and dorsal forearm. There was intermittent numbness, tingling, and burning in the same distribution and the right medial hand. Overall, 60% of his symptoms were in the chest wall and right upper limb. His pain level was 8/10. Sitting, standing, and driving for more than 20 minutes exacerbated his symptoms. Oddly, lying down aggravated his symptoms, especially in the chest wall and spreading to the other regions. There was no ideal position of comfort except putting his right hand behind his head, resulting in partial benefit. He denied any gait imbalance or bowel or bladder incontinence. Sleep was interrupted secondary to pain. He had weakness in his right hand and noticed some difficulty with dexterity such as handwriting.

Physical exam

Cervical spine motion was limited in all planes, especially in flexion. Cervical spine motion elicited right forearm and medial hand pain in flexion, medial arm pain with right lateral rotation, and right chest wall pain with right side bending. Cervical spine palpation exhibited tenderness over the right distal lateral masses. The right median nerve neural tension test was positive. Muscle bulk, tone, and strength were normal. Tactile sensation response in the right upper limb was decreased in the medial hand, including the fourth and fifth digits. Muscle stretch reflexes were normal without Hoffman’s sign. Gait was normal, and Romberg was negative.

Treatment

Given the location of the synovial cyst and the patient's presentation, a right C7-T1 laminoforaminotomy and synovial cyst resection surgery were recommended. An EMG was also recommended, but the patient declined and chose to proceed with surgery. While waiting, he tried Gabapentin, opioids, and oral non-steroidal anti-inflammatory drugs (NSAIDs), all of which provided partial relief. Uniquely, a taper of oral Prednisone twice resulted in the complete resolution of symptoms and functional limitations, lasting up to a few days after completion. Surgery was performed approximately three months after the initial presentation to our office. Following surgery, the patient had complete resolution of his symptoms and right-hand weakness. He was able to return to work and activities of daily living without restrictions and no longer required pain medications. He stated that he is *100% better and back to normal* and maintained improvement during the 19 months of follow-up.

## Discussion

Our case is unique compared to previously reported case reports or series because our patient's chest pain worsened when lying down. Cardiac or angina-based chest pain is known to worsen in the supine position because of increased venous return, resulting in increased volume load and oxygen demand [13].

Musculoskeletal-based or non-cardiac chest pain would be expected to improve with the supine position. Our hypothesis for this contradictory presentation is related to the relative neck flexion that occurred when lying down with the neck supported. A study by Reid [14] demonstrated that the lower cervical nerve roots are displaced during flexion, unlike the upper roots. In flexion, the elasticity of the spinal cord and dura leads to tightening of the spinal nerve roots and a slight inward shift. Based on the position of the synovial cyst in relationship to the right C8 nerve root, this small amount of motion would have a more dramatic impact. Synovial cysts are known to be adherent to the dura and the nerve root [7]. Thus, the motion is more likely to compress the C8 nerve root, as increased nerve root traction occurs due to the reduced ability to glide, given the presence of the synovial cyst. Also, with the synovial cyst narrowing the foraminal and central space, there would be less room for unopposed gliding of the nerve root, resulting in irritation.

Given that the C8 nerve root is compressed and more likely to be affected by small movements due to the synovial cyst, the question is: how can this manifest as chest pain? The C8 nerve root contributes to the median and/or lateral pectoral nerves. The median pectoral nerve, a pure motor nerve, supplies the pectoralis minor and pectoralis major muscles. The lateral pectoral nerve supplies the pectoralis major muscle and also carries proprioceptive and nociceptive fibers. The lateral pectoral nerve does connect to the medial pectoral nerve via the ansa cervicalis. Also, the median pectoral nerve may have protopathic sensibility. As a result, chest pain can manifest via referral through these peripheral nerves [15]. Second, the sinuvertebral nerve, a branch of each spinal nerve, passes in a recurrent pattern through the intervertebral foramen to supply the spinal meninges, posterior longitudinal ligament, posterolateral periphery of the intervertebral disc, and periosteum of the vertebrae. Thus, referred pain may be conducted via the sinuvertebral nerve [15]. As with other etiologies such as disc herniation or spondylosis, where the nerve root can be irritated and/or compressed, the result is symptoms like radiating pain in the distribution of peripheral nerves, in this case, the median and/or lateral pectoral nerves.

As for treatment, our patient did undergo a right C7-T1 laminoforaminotomy and synovial cyst resection. Practically, patients could entertain nonsurgical versus surgical care options, but the most common treatment is surgical resection. The most common surgical treatment is hemilaminectomy with cyst resection followed by decompressive laminectomy with cyst resection. In a study by Lyons et al., the largest published case series to date, all 35 patients were treated surgically.

## Conclusions

To our knowledge, our patient is only the seventh reported case of cervical hemorrhagic synovial cyst. Our case is unique because of the atypical presentation of nonanginal chest pain. Based on our case, cervical etiology for nonanginal chest pain would benefit from remaining on the differential until the completion of the diagnostic work-up.
